# ROCK (RhoA/Rho Kinase) in Cardiovascular–Renal Pathophysiology: A Review of New Advancements

**DOI:** 10.3390/jcm9051328

**Published:** 2020-05-02

**Authors:** Teresa M. Seccia, Matteo Rigato, Verdiana Ravarotto, Lorenzo A. Calò

**Affiliations:** 1 Department of Medicine, Hypertension Clinic, University of Padova, 35128 Padova, Italy; teresamaria.seccia@unipd.it; 2 Department of Medicine, Nephrology, Dialysis and Transplantation Unit, University of Padova, 35128 Padova, Italy; matteo.rigato@hotmail.it (M.R.); verdiana.ravarotto@gmail.com (V.R.)

**Keywords:** cardiovascular remodeling, kidney remodeling, ROCK, Rho, Rho kinase, hypertension, hypertensive nephropathy, posttransplant hypertension, Gitelman’s syndrome, Bartter’s syndrome

## Abstract

Rho-associated, coiled-coil containing kinases (ROCK) were originally identified as effectors of the RhoA small GTPase and found to belong to the AGC family of serine/threonine kinases. They were shown to be downstream effectors of RhoA and RhoC activation. They signal via phosphorylation of proteins such as MYPT-1, thereby regulating many key cellular functions including proliferation, motility and viability and the RhoA/ROCK signaling has been shown to be deeply involved in arterial hypertension, cardiovascular–renal remodeling, hypertensive nephropathy and posttransplant hypertension. Given the deep involvement of ROCK in cardiovascular–renal pathophysiology and the interaction of ROCK signaling with other signaling pathways, the reports of trials on the clinical beneficial effects of ROCK’s pharmacologic targeting are growing. In this current review, we provide a brief survey of the current understanding of ROCK-signaling pathways, also integrating with the more novel data that overall support a relevant role of ROCK for the cardiovascular–renal physiology and pathophysiology.

## 1. Introduction

The monomeric G protein RhoA and its target of activation, the Rho kinase (ROCK), comprise the RhoA/ROCK system that is important in a wide spectrum of physiological processes and pathways, e.g., contraction, focal adhesions, migration and proliferation. A prominent example of this is the signaling mediated by the RhoA/ROCK pathway and its interaction with angiotensin II (Ang II), oxidative stress and nitric oxide (NO), which play pivotal roles in the pathogenesis of cardiovascular diseases. The activation of RhoA/Rho kinase pathway acts first to increase calcium (Ca^2+^) sensitivity and vascular tone and then leads to cardiovascular–renal structural changes [1]. Recently, the increasing knowledge of the molecular mechanisms triggered by ROCK signaling has allowed us to gain a deeper insight into the pathogenesis of hypertension and cardiovascular–renal disease. Hence, this brief review will focus on the recent advancements in our understanding of the role/involvement of the RhoA/ROCK system in cardiovascular and renal pathophysiology.

## 2. ROCK1 and ROCK2

The two isoforms of Rho-associated coiled-coil-containing kinases, ROCK1 and ROCK2, are members of the serine/threonine AGC kinases family [2]. They are ubiquitously expressed, with high levels of ROCK1 found in non-neuronal tissues as spleen, lung, liver, kidney and testis, and ROCK2 detected mostly in the brain, muscles and heart.

**Figure 1:**
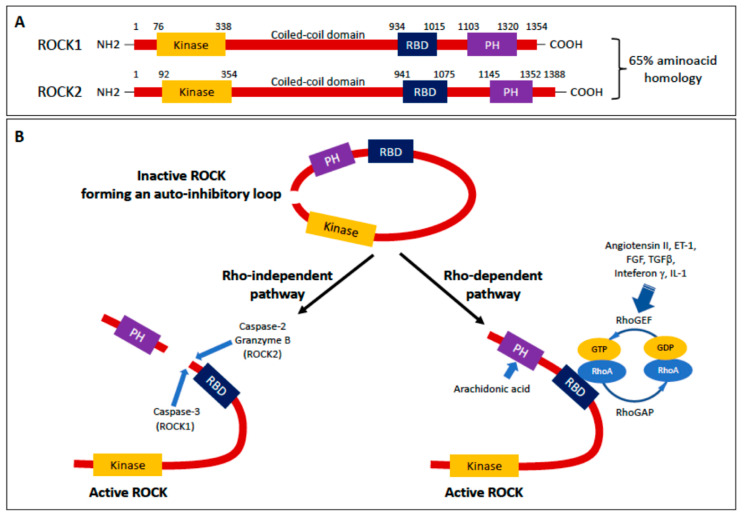
(**A**) Structure of Rho-associated coiled-coil containing kinase (ROCK) 1 and ROCK2. Both isoforms consist of a N-terminal kinase domain, a coiled-coil domain containing a Rho binding domain (RBD), a pleckstrin homology (PH) motif and a C-terminal cysteine-rich domain. ROCK1 and ROCK2 share 65% overall homology in amino acid sequence. (**B**) Inactivation/activation of ROCK. When the N-terminus is next to C-terminus, i.e., showing a closed configuration, the ROCK is inactive because of the auto-inhibitory loop. The opening of the loop can be caused 1) by caspase-3, or caspase-2 and granzyme B, that cleave the C-terminus of ROCK1 and ROCK2, respectively, or 2) by a Rho-dependent pathway, via binding of GTP-RhoA to the RBD, driven by angiotensin II, endothelin (ET) 1, fibroblast growth factor (FGF) and TGFβ, interleukin (IL)-1 or interferon γ. Modified from Hartmann, S., et al. [2] and Shimizu, T., et al. [3].

The structure of ROCK1/2 encompasses three regions: (1) an N-terminal region with the kinase domain, (2) a central coiled-coil region with the Rho-binding domain (RBD) followed by (3) a C-terminal region comprising the pleckstrin homology (PH) motif (Figure 1) [3]. The crystal structure of ROCK exhibits a hook in the coiled-coil central region and two dimerization domains in the N-terminus region. These features allow the N-terminal kinase domains to interact with the protein’s C-terminus inhibitory region forming a homodimer that, under basal conditions, inactivates the kinase being in a ‘closed’ (*inactive*) conformation. Upon binding of Rho A to RBD, ROCK’s loop ‘opens’, exposing the N-terminal kinase domain and activating the N-terminal kinase (Figure 1). However, activation of ROCK can also occur by binding arachidonic acid to PH domain, or by cleavage of the C-terminus induced by caspase-2 or -3, or granzyme B (Figure 1) [4,5].

RBD maintains high homology, which accounts for equal affinity to RhoA, RhoB and RhoC, whereas coiled-coil domains and C-terminal PH region sequences differ between ROCK1 and ROCK2, leading to phosphatidylinositol [3,4,5] triphosphate (PIP_3_) and phosphatidylinositol [4,5] biphosphate (PIP_2_), preferably binding ROCK2 but not ROCK1. Such differences also explain why caspase-3 cleaves the C-terminus in ROCK1, which contains the DETD1113/G sequence, but not cleaving ROCK2 that, in contrast, lacks such a sequence. Moreover, the poor homology in C-terminus PH region might also explain the different localization of the two ROCK isoforms in the cell, with ROCK2 being associated with centrosome and actin in the nucleus and cytosol, and ROCK1 primarily associated with the plasma membrane and vesicles [2].

## 3. Mechanisms of ROCK Regulation

One of the upstream signals for RhoA/Rho kinase pathway is the binding of Ang II or other peptides as Endothelin (ET)-1, fibroblast growth factor (FGF) and transforming growth factor (TGF) β to their receptors on the cell membrane [6]. Other stimuli can come from interleukin (IL)-1 and interferon γ (Figure 1). These then act through their receptors G-coupled proteins to mobilize the Gα subunit of the Gq protein, which activates Rho-guanine exchange factors (RhoGEFs) to replace guanosine-5′-diphosphate (GDP) with GTP (Figure 1) [7]. As mentioned above, the GTP-RhoA interaction with the RBD of the ‘closed’ homodimer promotes ROCK kinase activity via a *derepression* mechanism, where the repression of the kinase domain by the C-terminus is removed leading to the active ‘open’ conformation. It is not clear whether this mechanism is entirely responsible for the activation or requires additional phosphorylation in other sites [2]. Among the GEFs, p63RhoGEF and p115RhoGEF are mediators of Ang II, allowing downstream activation of AT1R to ROCK1/2 via RhoA protein [1,8]. Activation of RhoA/GTP is turned off by GTPase-activating proteins (RhoGAPs) that induce hydrolysis of GTP to GDP (Figure 1).

Mechanisms of activation of ROCK1 specifically involve caspase 3-mediated removal of the auto-inhibitory C-terminus at the DETD1113/G sequence, which occurs in the execution phase of apoptosis, leading to membrane blebbing. Changes in cell morphology characteristic of blebbing are associated with increased myosin light chain (MLC) phosphorylation by a Ca^2+^/calmodulin-dependent kinase and by ROCK-mediated phosphorylation of the phosphatase subunit of MLC, the myosin phosphatase subunit target (MYPT)-1 [9].

However, there are data that suggest more involvements of ROCK1, as in cardiac fibrosis [10,11], leukocyte recruitment and neointima formation following vascular injury [12], deterioration of ventricular function [13], and also arrhythmias [14].

Despite being not susceptible to caspase-3 because it lacks the DETD1113/G sequence, ROCK2 has a cleavage site for the protease granzyme B and the caspase-2 [5,15] upstream of the PH domain. ROCK2 signaling was found to be involved in arterial hypertension, atherosclerosis, myocardial hypertrophy and ischemia-reperfusion injury, vascular remodeling and stroke [3]. Moreover, gene polymorphisms in the ROCK2 were found to be associated with different risk of developing hypertension [12], and ROCK2 activity was found to modulate the circadian blood pressure variations under the influence of brain and muscle aryl hydrocarbon receptor nuclear translocator-like (BMAL1) clock gene [16]. BMAL1, by a time-of-day-variation of directly binding the promoter of ROCK2, could enhance myosin phosphorylation and vasoconstriction [16]. Via upregulation of the RhoA protein and prevention of its degradation, BMAL1 could further boost ROCK’s mediated reorganization of stress fibers and formation of actin cytoskeleton, reinforcing vasoconstriction [17]. 

## 4. Downstream Targets of ROCKS

**Figure 2:**
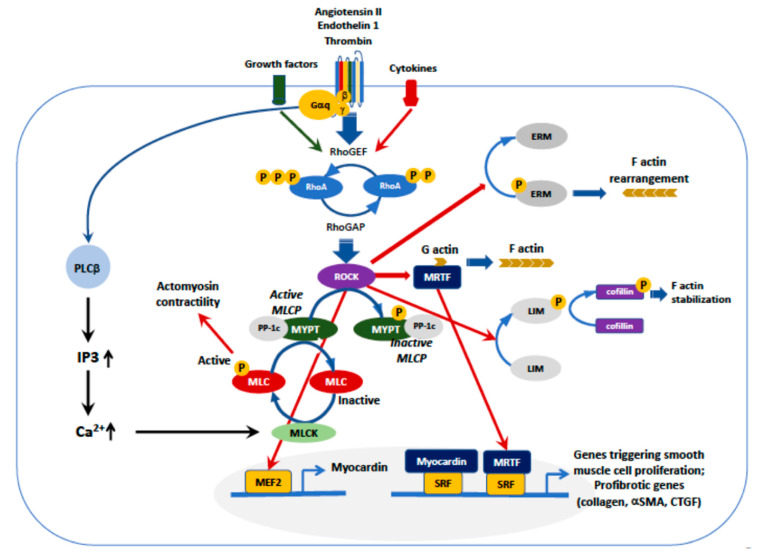
Upstream and downstream regulation of ROCK. Activation of G protein-coupled receptors, e.g., AT1 or ET-1, or receptors for cytokines or other growth factors, activate RhoGEF that, in turn phosphorylates RhoA/ROCK. The major ROCK downstream pathways are (1) myosin light chain phosphatase (MLCP)/MLC, (2) ezrin, radixin and moesin (ERM), (3) myocardin and serum response factor (SRF), (4) LIM (Lin-11, Isl-1,Mec-3))/cofilin, (5) assembly of F-actin with release of myocardin-related transcription factor (MRTF). The assembly of F-actin enables MRTF to dissociate from G-actin, leading to MRTF nuclear translocation and binding to SRF, which triggers transcription of genes involved in cardiovascular remodeling. However, all pathways concur to the development of smooth muscle cell proliferation, transition of myocytes into myofibroblasts and fibrosis, or stress fibers formation.

As noted above, a major target of ROCK is MYPT-1. Phosphorylation of MYPT-1 by ROCK leads, via inhibition of the myosin light-chain phosphatase (MLCP), to persistent MLC phosphorylation (Figure 2). The inhibition of MLCP promotes actomyosin-based contractility which contributes to stress fiber formation, Ca^2+^ sensitization of the vascular smooth muscle cells (VSMCs) and contraction [18].

ROCK-mediated Ca^2+^ sensitization is also crucial for the regulation of myocyte enhancer factor (MEF)-2-dependent expression of myocardin, a specific transcriptional coactivator of serum response factor (SFR), which controls cell proliferation (Figure 2) [19]. The role of myocardin in proliferation has been supported by tumor growth after its inactivation [20]. Through the MEF2 pathway, ROCK has been shown to play a significant role in the phenotypic modulation of VSMCs, acting in the regulation of early genes involved in proliferation and migration [3,21]. High levels of myocardin mRNA were found in circulating cells and in the cardiac tissue from patients with essential hypertension and ventricular hypertrophy, suggesting a role in the cardiac damage as well [22,23,24]. Moreover, Tang et al. found that nuclear factor kappa-light-chain enhancer of activated B cells (NF-κB), a transcription factor involved in cellular proliferation, can suppress myocardin activity [20]. Activation of NF-κB follows the degradation of its inhibitory subunit IκB, which occurs through phosphorylation via the RhoA-Rho kinase system. Hence, ROCK favors NF-κB activation that, in turn, blunts myocardin activity, finally leading to cell proliferation. 

Notably, increased mononuclear cells IκB levels have been reported in Bartter’s and Gitelman’s syndromes that, being models of endogenous Ang II signaling antagonism, represent the hypertension’s mirror image [25]. The decreased IκB levels were supposed to be linked to the reduced activity of RhoA/Rho kinase system observed in these patients [1,26,27]. 

Other downstream targets of ROCK, as ezrin, radixin and moesin (ERM), are involved in actin filament/plasma membrane interactions, which play a major role in endothelial (dys)function and inflammation (Figure 2) [28]. ERM proteins integrate Rho GTPase signaling as upstream regulators or downstream targets [29]. Phosphorylation of moesin’s threonine residue by ROCK is critical for ERM activation, which mediates cytoskeletal rearrangement and endothelial permeability. To activate ERM proteins, ROCK pathway can act in concert with reactive oxygen species (ROS), NADPH oxidases and advanced glycation end products (AGEs) [30,31,32].

ROCK, by phosphorylating LIM kinase, induces phosphorylation of cofilin (Figure 2) that stabilizes actin filaments [33]. In a cofilin-2 knock out mouse model, an increase in the left ventricular mass was found along a decrease in wall thickness and contractile function, a condition that resembles dilated cardiomyopathy [34].

## 5. ROCKs in Cardiovascular and Renal Remodeling

Cardiovascular events are the major clinical challenges in populations with hypertension and/or chronic kidney disease (CKD) [35,36].

Uncontrolled or unresolved activation of the RhoA/Rho kinase pathway was found to be associated with cardiovascular and renal remodeling [1,18,37]. ROCK activation induces expression of proinflammatory cytokines, adhesion molecules, and of atherothrombogenic plasminogen activator inhibitor (PAI)-1, and furthermore, regulates cyclophilin A, a chaperon protein originally identified as the target of the immunosuppressive drug cyclosporine and then found to act as a secreted oxidative stress-induced factor that promotes inflammation [38].

ROCK phosphorylates and inhibits phosphatase and tensin homology (PTEN), thus blocking the pro-survival phosphoinositide 3-kinase (PI3K) pathway [39]. Moreover, since the PI3K/Akt pathway promotes the expression of endothelial nitric oxide synthase (eNOS), ROCK-mediated activation of PTEN leads to decreased nitric oxide (NO) production and reduced cell survival of endothelial cells [39]. 

The balance between RhoA/Rho kinase pathway and NO system plays a crucial role in the cardiovascular and renal dysfunction, providing induction/decrease of oxidative stress and inhibition/activation of PI3K/Akt [40]. The production of superoxide anion (O_2_^−^) from NO results in endothelial dysfunction and accumulation of cytosolic Ca^2+^ that could affect myocardial relaxation and contractility [41]. The alteration of Ca^2+^ handling by sarcoplasmic reticulum as a consequence of ROCK activity and induction of oxidative stress, also increases the expression of Ca^2+^/calmodulin-dependent kinase II (CaMKII), a regulator of excitation/contraction coupling. Changes in CaMKII also increase the vascular tone and sodium influx via voltage-gated channels, with prolongation of the action potential and possibly of arrhythmias [42,43]. NADPH oxidases (Nox), which are ROS generating enzymes activated by vasoactive peptides as Ang II and ET-1, may represent a crosstalk in this scenario between calcium and ROS, and likely also ROCK [44].

In dialysis and CKD patients, the ROCK pathway was found to be overactive, particularly in patients who already had left ventricular hypertrophy [45,46]. After exposure to the Rho kinase inhibitor fasudil, leukocytes isolated from patients with CKD and/or dialysis, showed a dose dependent reduction of ROCK activity, suggesting that modulation of ROCK could be useful for prevention of cardiovascular–renal remodeling [46].

Both acute and chronic kidney diseases are featured by spotted or diffuse changes of tubular epithelial cells that can lead to progressive and potentially irreversible renal failure [47]. Such alterations are mediated by changes in F-actin structure, primarily regulated by the activity of GTPases of the Rho family. 

The critical role of the ROCK pathway in renal and cardiovascular remodeling entails oxidative stress and oxidative stress-related signaling through induction of NADPH oxidase [48]. The production of ROS from NADPH is under the control of the cytochrome b_558_, and in particular of the subunit p22^phox^ that is essential for the electron transport from NADPH to haeme and its transfer to the molecular oxygen, generating O_2_^−^ [49,50]. One of the most important effects of ROS is the reduction of NO bioavailability via downregulation of eNOS and O_2_^−^ production, by reacting with NO, turns it into peroxinitrite thereby reducing NO bioavailability [25,51]. Of note, an increased p22^phox^ protein expression was found in patients with CKD, end stage renal disease, dialysis patients with left ventricular hypertrophy, patients with primary (essential) hypertension, Fabry disease, renal transplantation, and primary hyperaldosteronism [52,53,54,55,56]. 

RhoA/Rho kinase also participates to C-reactive protein (CRP)-induced atherothrombogenesis. CRP can promote NF-κB activity through ROCK pathway, resulting in PAI-1 expression whose activity is crucial for cytokinesis, cell migration and invasion [1]. 

ROCK involvement in the cardiovascular and renal remodeling occurs via either direct or indirect pathways. Directly, this occurs through phosphorylation of ROCK substrates, as MYPT-1 that is connected to vasoconstriction, or ERM and LIM, which are connected with actin filaments and cytoskeletal rearrangement, and indirectly through altered levels of redox active molecules as O_2_^−^. The responses induced by oxidative stress comprise the activation of Extracellular signal-Regulated Kinases (ERK) 1/2 and Mitogen-Activated Protein Kinases (MAPK). Under the control of cellular stimuli that converge on GTPase proteins as Ras and Rap1, a phosphoryl group (PO_3_^2−^) is transferred to tyrosine and threonine groups of ERK1/2. The activation of ERK1/2 induces an integrated response that, by triggering nucleotide and protein synthesis, activates transcription factors and chromatin phosphorylation [57] and, by enhancing Ca^2+^ availability, ultimately causes cell contraction over and above that directly induced by ROCK [58].

**Figure 3:**
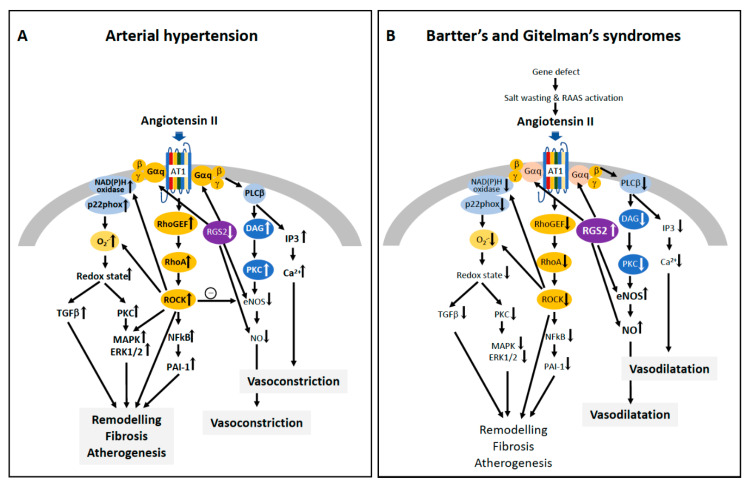
Schematic representation of Angiotensin II-mediated pathways involving RhoA/ROCK in arterial hypertension (**A**) and Bartter’s and Gitelman’s syndromes (**B**). Angiotensin II, via angiotensin II type 1 receptor (AT1R), activates the cascade RhoGEF/RhoA/ROCK, which leads to vascular remodeling, cardiac and renal fibrosis, and atherogenesis in hypertension (Panel A). The sketch reports the NF-κB/PAI-1 and NADPH oxidase/ superoxide anion (O_2_^−^) as representative ROCK downstream pathways, but other pathways can be involved (see Figure 2). AT1-mediated pathways also involve mitogen-activated protein kinases (MAPK and ERK 1/2), diacylglycerol (DAG)/protein kinase C (PKC) and inositol triphosphate (IP3), which leads to increased Ca^2+^ release in the cytosol with vasoconstriction. In Bartter’s and Gitelman’s syndromes (Panel B), despite increased levels of Angiotensin II, the pathways leading to vasodilatation prevail on those leading to vasoconstriction due to post receptor abnormalities including blunted Gαq protein signaling and activation of the regulatory G protein signaling 2 (RGS2). RGS2 causes activation of endothelial nitric oxide synthase (eNOS) with increased Nitric Oxide (NO) release and blunted ROCK activity and signaling, with reduced intracellular Ca^2+^ sensitivity finally leading to vasodilation. Modified from Calò, L.A. et al. [1,25].

Increased ROCK activity, measured in terms of increased p63 RhoGEF protein level and increased phosphorylation of MYPT-1, increased levels of p22^phox^ and ERK1/2, as well as increased oxidated low density lipoproteins (oxLDL), were found in patients with hypertension (Figure 3, Panel A), CKD and end stage renal disease on dialysis who had left ventricular hypertrophy, supporting the role of ROCK and ERK1/2 signaling in the cardiovascular–renal remodeling [26,46,59,60]. This was further supported by the reduced ROCK activity, reduced oxidative stress and ERK1/2 protein expression along lack of hypertension and cardiovascular–renal remodeling in patients with Bartter’s and Gitelman’s syndromes (Figure 3B) [8].

Oxidative stress and ROCK signaling also play a role in Fabry’s disease, a rare genetic disease characterized by deficient activity of α-galactosidase A and progressive accumulation of globotriaosylceramide (Gb3) and LysoGb3 in lysosomes. This accumulation can lead to cardiac infarction, heart failure and chronic kidney disease [61]. Despite the availability and use of α-galactosidase A enzyme replacement therapy, Fabry’s patients still exhibit serious myocardial, cerebral and renal abnormalities. In Fabry patients, ROCK activity, as indicated by MYPT-1 phosphorylation levels, was found to be higher than in healthy subjects, along increased expression of p22^phox^ and the lipid peroxidation marker malondialdehyde, and also higher left ventricular mass. 

Moreover, a recent study suggested a role of ROCK also in atrial fibrillation [62]. ROCK activity, measured as MYPT-1 phosphorylation, was found to be increased in dialysis patients with atrial fibrillation compared to dialysis patients without atrial fibrillation [62], and to correlate with the expression of connexin40 (Cx40), a membrane protein relevant for rapid cell–cell transfer of action potential in the heart, as well as with the left atrial systolic volume and the left ventricular mass, again identifying a connection between ROCK activity and cardiovascular–renal remodeling [63]. 

## 6. ROCK Inhibition

Transgenic animal models with ROCK deletion suggest an important role of this kinase in cardiovascular–renal remodeling [13,64]. In a model of Gαq overexpression, ROCK1 deletion attenuated left ventricular dilation and cardiomyocyte apoptosis, but did not prevent the development of cardiac hypertrophy. In addition, mimicking pressure overload-induced hypertrophy in cardiomyocytes with overexpression of Gαq and homozygous knockout of ROCK1, a decreased expression of profibrotic factors as TGFβ2 and CTGF, as well as reduced left ventricular dilation and cardiomyocyte apoptosis, were found, but no prevention of cardiac hypertrophy was observed [11,65]. In contrast, the overexpression of ROCK1 promoted cardiomyocytes apoptosis and cardiomyopathy [66]. 

A positive feed-forward regulatory loop of ROCK1 has been contended: when the cleaved constitutively active form is accumulated in cardiomyocytes, it leads to caspase-3 activation [13,67]. Then, ROCK1-dependent caspase-3 activation could push PTEN signaling to inhibit PI3K/Akt and eNOS. 

A human model of the endogenous blockade of ROCK signaling comes from Bartter’s and Gitelman’s syndromes. These patients have blunted Ang II signaling along decreased expression of the α subunit of Gq protein and blunted downstream intracellular events, as reduced intracellular Ca^2+^ release and PKC activation (Figure 3, Panel B) [25,68,69]. 

Bartter’s and Gitelman’s patients, notwithstanding high circulating levels of Ang II and aldosterone, display normal or low blood pressure and are protected by cardiovascular remodeling and adverse cardiovascular events [25]. We have shown that Gαq is downregulated as a consequence of increased expression of regulator of G protein signaling (RGS)-2, which is also crucial for the vasodilatory activity of NO [70,71]. In these patients, the net balance between RhoA/Rho kinase pathway and NO is favorable to antioxidant defenses, as both NO and eNOS are increased and RhoA/ROCK signaling is downregulated [72]. This is supported by the findings that p63RhoGEF gene and protein expression, which selectively responds to Ang II to activate RhoA/ROCK pathway, is reduced along with the reduction of MYPT-1 phosphorylation, while the opposite was observed in essential hypertensive patients [8,26].

The pharmacological targeting of ROCK has been evaluated in rats infused with Ang II: the simultaneous treatment with fasudil, a ROCK inhibitor, significantly suppressed the Ang II-induced left ventricular hypertrophy and cardiomyocyte hypertrophy [31]. Y-27632, another ROCK inhibitor, suppresses RhoA protein and consequently ROCK gene expression and MYPT-1 phosphorylation. In Dahl salt-sensitive rats, fasudil prevented the increase of left ventricular weight, while in hypertensive rats the treatment attenuated myocardial fibrosis [73]. In rats with pressure overload hypertrophy, selective inhibition of ROCK with GSK-576371 improved left ventricular geometry, collagen deposition and diastolic function [74]. 

Left atrial enlargement, interstitial fibrosis and hypertension are common in CKD and are thought to result in the increased prevalence of atrial fibrillation in CKD and dialysis patients [75]. Recently, we evaluated the effects of fasudil in the peripheral blood mononuclear cells from dialysis patients with atrial fibrillation. We found increased ROCK activity and overexpression of connexin 40, which correlated with left atrial volume and ventricular mass. Fasudil treatment reduced not only MYPT-1 phosphorylation, but also connexin 40, suggesting a link between ROCK activity and atrial remodeling. 

## 7. ROCK in Hypertensive Nephropathy and Post-Transplant Hypertension

Uncontrolled and chronically elevated blood pressure triggers hypertensive nephropathy, which results in vascular, tubule-interstitium and glomerular damage, with hyperfiltration/hypertrophy of the nephrons finally leading to renal failure [76]. The activation of ROCK by upstream signals as Ang II or ET-1, together with decreased NO bioavailability, is the boost to induction of tubule-interstitial fibrosis, podocyte loss and epithelial-to-mesenchymal transition (EMT) of tubular epithelial cells [32], these latter proposed as emerging mechanisms of the hypertensive nephropathy [76]. In a transgenic rat model of severe hypertension and cardiovascular damage, either the blockade of type 1 Ang II receptor (AT1R) or the endothelin system were able to prevent tubule-interstitial fibrosis [77,78].

Kidney transplant recipients undergo immunosuppressive therapy with calcineurin inhibitors, cyclosporine (CsA) and tacrolimus [48], which lead to vasoconstriction and dysregulation of kidney sodium transport. This occurs mainly through the activation of the renin-angiotensin system [48] alongside an increased expression of oxidative stress-related proteins and decreased anti-oxidant defenses [53,79]. The CsA-induced up-regulation of Ang II increases intracellular Ca^2+^ and calcium sensitivity via ROCK, whose activity has been shown to be blunted by calcineurin via inhibition of MYPT-1 phosphorylation. Overall, these findings suggest that ROCK inhibition could be helpful to modulate the impact of calcineurin and its inhibitors by contrasting smooth muscle contraction and hypertension [80].

## 8. Clinical Implications

Given the deep involvement of ROCK in cardiovascular–renal pathophysiology and the interaction of ROCK signaling with other signaling pathways, the reports of trials on the clinical beneficial effects of ROCK’s pharmacologic targeting are growing, and not only for cardiovascular disease [1]. ROCK inhibition, in fact, has shown promising clinical effects as a therapeutic target in a variety of human diseases including, in particular, cardiovascular (angina, heart failure, hypertension, pulmonary hypertension), renal (renoprotection in renal disease, chronic kidney disease) and diabetes [10,81], but also a clinical interest is developing for medical therapies of cerebrovascular diseases, glaucoma and autoimmune diseases [1,10,81].

The development of more translational research will further help a clear and complete comprehension of the role of ROCK in human physiology, pathophysiology and diseases, while the need for more clinical trials with ROCK inhibitors in human diseases where they, driven by a clearer knowledge of ROCK pathophysiology, might be efficiently used, is required to assess their clinical usefulness in human disease.

## 9. Conclusions and Future Perspectives

The renin–angiotensin–aldosterone signaling system plays a pivotal role in the biochemical/molecular mechanisms which underlie cardiovascular–renal disease. ROCK, as downstream mediator of Ang II signaling, represents a major inducer of cardiac–renal remodeling. The centrality of ROCK signaling in this process then leads to ROCK inhibition as a key target for the protection/prevention of cardiovascular–renal disease. However, given the multiplicity of roles it plays, more studies are needed to ensure that ROCK inhibition produces important beneficial effects with no relevant side effects. However, based optimistically on our current understanding, the development of ROCK inhibitors will likely yield cardiovascular–renal protection comparable to that resulted over the last three decades by the development of ACE inhibitors, AT1R blockers, and aldosterone receptor antagonists.
